# Phase I trial of SPH4336, a novel cyclin-dependent kinase 4/6 inhibitor, in patients with advanced solid tumors

**DOI:** 10.1093/oncolo/oyaf077

**Published:** 2025-06-30

**Authors:** Yu Jiang, Xu Liang, Mei-Li Sun, Ge Gao, Yi Gong, Hui-Ping Li, Jie Liu, Yong-Sheng Wang

**Affiliations:** Cancer Center, West China Hospital of Sichuan University, Chengdu, Sichuan, 610041, People’s Republic of China; Key Laboratory of Carcinogenesis and Translational Research (Ministry of Education/Beijing), Department of Breast Oncology, Peking University Cancer Hospital & Institute, Beijing, 100142, People’s Republic of China; Jinan Central Hospital, Shandong University, Central Hospital Affiliated to Shandong First Medical University, Jinan, Shandong, 250013, People’s Republic of China; Cancer Center, West China Hospital of Sichuan University, Chengdu, Sichuan, 610041, People’s Republic of China; Chongqing University Cancer Hospital, Chongqing, 400030, People’s Republic of China; Key Laboratory of Carcinogenesis and Translational Research (Ministry of Education/Beijing), Department of Breast Oncology, Peking University Cancer Hospital & Institute, Beijing, 100142, People’s Republic of China; Cancer Center, West China Hospital of Sichuan University, Chengdu, Sichuan, 610041, People’s Republic of China; Cancer Center, West China Hospital of Sichuan University, Chengdu, Sichuan, 610041, People’s Republic of China

**Keywords:** cyclin-dependent kinase 4/6 inhibitor, advanced solid tumours, first-in-human trial, SPH4336

## Abstract

**Background:**

Preclinical models demonstrated promising anti-tumor activity of SPH4336, a novel oral, highly selective cyclin-dependent kinase (CDK) 4/6 inhibitor.

**Methods:**

This phase I study enrolled patients who received SPH4336 orally in 6 dose-escalation cohorts (50-600 mg) in a 3 + 3 design. Based on tolerability, pharmacokinetics (PK) and activity data from the dose-escalation phase, 2-3 dose cohorts were expanded. Dose-limiting toxicity (DLT), maximum tolerated dose (MTD), recommended phase II dose (RP2D), efficacy, safety, tolerability, and pharmacokinetics (PK) were investigated.

**Results:**

A total of 29 patients with breast cancer (BC) (*n* = 14), sarcoma (*n* = 8), non-small cell lung cancer (*n* = 2) and others (*n* = 5) were enrolled. Neither DLT nor MTD were reached. All patients had at least one treatment-related adverse events (TRAEs), most of which were grade 1/2. Grade ≥ 3 TRAEs occurred in 51.7% of patients. One patient died from disease progression and five reported serious adverse events. Plasma concentrations increased dose-dependently, except at 600 mg, and steady state was reached at 2 weeks for 400 mg. One BC patient in the 600-mg cohort had a confirmed partial response. The disease control rate was 59.3% (95% CI, 38.8-77.6).

**Conclusion:**

SPH4336 demonstrated an acceptable safety profile and dose-dependent plasma exposure in patients with various advanced solid tumors. (ClinicalTrials.gov Identifier: NCT05905614; IRB Approved.)

**Lessons learned**

- This first-in-human phase I study of SPH4336, a novel oral cyclin-dependent kinase (CDK) 4/6 inhibitor, demonstrated potential impact on advanced solid tumours.
- A favorable safety profile and no dose-limiting toxicities were observed.
- Preliminary efficacy showed a confirmed partial response in a patient with breast cancer and an overall disease control rate of 59.3%. PFS exceeds 6 months in patients with sarcoma and breast cancer.

## Discussion

This phase I trial evaluated the safety, tolerability, PK, and preliminary anticancer activity of SPH4336, a novel selective CDK4/6 inhibitor, in patients with advanced solid tumors. TRAEs were observed in 29/29 (100%) of patients with 15/29 (51.7%) having grade ≥3 TRAEs. The most frequent haematologic TRAEs were decreased leucocyte count (*n* = 21, 72.4%), decreased neutrophil count (*n* = 20, 69.0%), and anemia (*n* = 19, 65.5%). The most frequent non-haematologic TRAEs were diarrhea (*n* = 20, 69.0%), vomiting (*n* = 12, 41.4%), abdominal pain, and nausea (*n* = 9 each, 31.0%). The most frequent grade ≥3 TRAEs included decreased leucocyte, lymphocyte count (*n* = 4 each, 13.8%) and decreased neutrophil count (*n* = 3, 10.3%) (**Table 1**). Neither DLT events were observed nor MTD was reached up to the highest preset dose of 600 mg.

**Table 1.** TRAEs occurring in ≥ 10% of patients treated with SPH4336 QD (graded according to the National Cancer Institute Common Terminology Criteria for Adverse Events, version 5.0).

		50 mg	100 mg	200 mg	300 mg	400 mg	600 mg	All
**TRAE, *n* (%)**	**Grade**	***N* = 3**	***N* = 3**	***N* = 3**	***N* = 4**	***N* = 9**	***N* = 7**	***N* = 29**
All TRAEs	All	3 (100)	3 (100)	3 (100)	4 (100)	9 (100)	7 (100)	29 (100)
	≥3	2 (66.7)	0	1 (33.3)	1 (25.0)	7 (77.8)	4 (57.1)	15 (51.7)
Haematologic AEs								
Leucocyte count decreased	All	1 (33.3)	1 (33.3)	1 (33.3)	4 (100)	8 (88.9)	6 (85.7)	21 (72.4)
	≥3	0	0	0	0	2 (22.2)	2 (28.6)	4 (13.8)
Neutrophil count decreased	All	1 (33.3)	1 (33.3)	1 (33.3)	4 (100)	7 (77.8)	6 (85.7)	20 (69.0)
	≥3	0	0	0	0	2 (22.2)	1 (14.3)	3 (10.3)
Anaemia	All	2 (66.7)	1 (33.3)	2 (66.7)	2 (50.0)	5 (55.6)	7 (100)	19 (65.5)
	≥3	1 (33.3)	0	0	0	0	0	1 (3.4)
Lymphocyte count decreased	All	1 (33.3)	1 (33.3)	3 (100)	3 (75.0)	2 (22.2)	2 (28.6)	12 (41.4)
	≥3	1 (33.3)	0	1 (33.3)	1 (25.0)	0	1 (14.3)	4 (13.8)
Platelet count decreased	All	1 (33.3)	0	0	2 (50.0)	4 (44.4)	0	7 (24.1)
	≥3	0	0	0	0	0	0	0
Nonhematologic AEs								
Diarrhoea	All	1 (33.3)	2 (66.7)	1 (33.3)	3 (75.0)	7 (77.8)	6 (85.7)	20 (69.0)
	≥3	0	0	0	0	2 (22.2)	0	2 (6.9)
Vomiting	All	0	0	0	0	6 (66.7)	6 (85.7)	12 (41.4)
	≥3	0	0	0	0	0	0	0
Abdominal pain	All	0	0	0	3 (75.0)	3 (33.3)	3 (42.9)	9 (31.0)
	≥3	0	0	0	0	0	0	0
Nausea	All	0	0	1 (33.3)	0	5 (55.6)	3 (42.9)	9 (31.0)
	≥3	0	0	0	0	1 (11.1)	0	1 (3.4)
γ-glutamyl transpeptidase increased	All	1 (33.3)	1 (33.3)	0	1 (25.0)	2 (22.2)	2 (28.6)	7 (24.1)
	≥3	0	0	0	0	1 (11.1)	1 (14.3)	2 (6.9)
Hypokalaemia	All	0	0	0	0	4 (44.4)	3 (42.9)	7 (24.1)
	≥3	0	0	0	0	2 (22.2)	0	2 (6.9)
Aspartate aminotransferase increased	All	0	0	0	0	4 (44.4)	2 (28.6)	6 (20.7)
	≥3	0	0	0	0	0	0	0
Fatigue	All	0	0	0	0	3 (33.3)	2 (28.6)	5 (17.2)
	≥3	0	0	0	0	2 (22.2)	0	2 (6.9)
Alanine aminotransferase increased	All	0	0	0	0	3 (33.3)	1 (14.3)	4 (13.8)
	≥3	0	0	0	0	1 (11.1)	0	1 (3.4)
Hyponatraemia	All	0	0	1 (33.3)	0	1 (11.1)	2 (28.6)	4 (13.8)
	≥3	0	0	0	0	0	0	0
Dizziness	All	2 (66.7)	0	0	0	0	2 (28.6)	4 (13.8)
	≥3	0	0	0	0	0	0	0
Alkaline phosphatase increased	All	1 (33.3)	1 (33.3)	0	0	1 (11.1)	1 (14.3)	4 (13.8)
	≥3	0	0	0	0	0	0	0
Hypoalbuminemia	All	0	0	2 (66.7)	1 (25.0)	0	1 (14.3)	4 (13.8)
	≥3	0	0	0	0	0	0	0
Appetite decreased	All	0	0	1 (33.3)	1 (25.0)	0	2 (28.6)	4 (13.8)
	≥3	0	0	0	0	0	0	0
Blood lactic dehydrogenase increased	All	0	0	0	0	1 (11.1)	2 (28.6)	3 (10.3)
	≥3	0	0	0	0	0	0	0
Rash	All	1 (33.3)	0	0	0	1 (11.1)	1 (14.3)	3 (10.3)
	≥3	0	0	0	0	0	0	0
Electrocardiogram QT prolonged	All	0	2 (66.7)	1 (33.3)	0	0	0	3 (10.3)
	≥3	0	0	0	0	0	0	0
Blood creatinine increased	All	0	1 (33.3)	0	1 (25.0)	1 (11.1)	0	3 (10.3)
	≥3	0	0	0	0	0	0	0
Haematuria	All	1 (33.3)	1 (33.3)	0	1 (25.0)	0	0	3 (10.3)
	≥ 3	0	0	0	0	0	0	0

**Figure 1.**
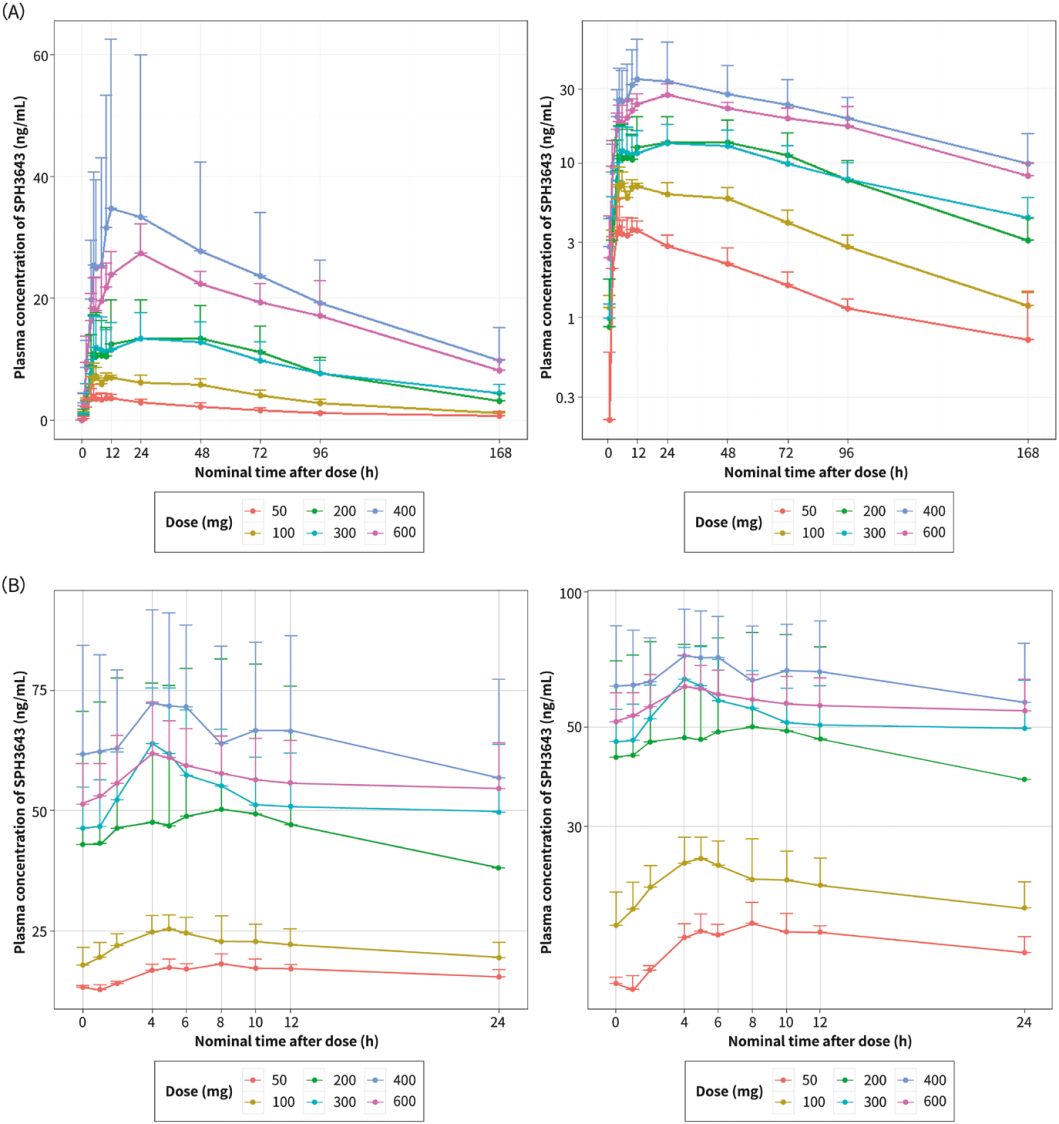
The mean plasma concentration-time profile of SPH3643 after (A) single dosing and (B) multiple dosing of SPH3643.

SPH4336 plasma exposure increased proportionally within the range of 50-400 mg, except at 600 mg, suggesting absorption saturation at 400 mg once daily (QD) (**Figure 1**). The RP2D of SPH4336 was established as 400 mg, at which SPH4336 effectively inhibited CDK4 and CDK6 activity and showed promising antitumor activity.

All patients included in this study had advanced solid tumors refractory or intolerable to current standard therapies. Preliminary antitumor activity of SPH4336 was observed at the initial dose of 50 mg, with all 3 patients achieving stable disease. At the 600 mg dose, 1 patient achieved partial response, resulting in an overall objective response rate of 3.7%. Patients appeared to benefit from the investigational treatment, with an overall disease control rate of 59.3% (95% CI, 38.8%-77.6%) and a median progression-free survival (PFS) of 3.1 months (95% CI, 1.7-5.7). An analysis of PFS suggested that SPH4336 may be promising in the treatment of breast cancer (BC) and sarcoma, with PFS exceeding 6 months in 6 patients (50 mg, *n* = 1, liposarcoma; 100 mg, *n* = 1, chondrosarcoma; 400 mg, *n* = 2, both BC; 600 mg, *n* = 2, BC and liposarcoma).

In conclusion, this study demonstrates that SPH4336 is well tolerated and safe within the dose range from 50 to 600 mg QD. Ongoing phase I/II clinical trials are further investigating the therapeutic effects and safety profile of SPH4336 in various advanced solid tumors (NCT05744687, NCT05860465, NCT05872347, NCT05580588, and NCT05944224).

**Table:** 

Trial Information	
Disease	Advanced cancer/solid tumor
Stage of Disease	Advanced
Prior Therapy	No designated number of regimens
Study Type	Interventional
Study Phase	Phase 1
Primary Endpoint	Maximal tolerated dose (MTD)
Primary Endpoint	Dose-limiting toxicity (DLT)
Secondary Endpoint	Objective response rate (ORR)
Secondary Endpoint	Progression-free survival (PFS)
Secondary Endpoint	Pharmacokinetics parameters
Secondary Endpoint	Disease control rate (DCR)
Secondary Endpoint	Duration of remission (DOR)
Secondary Endpoint	Safety and tolerability
Study Design	Allocation: *N*/A Interventional Model: Single group assignment Masking: None (Open Label) Primary Purpose: Treatment
Drug Information For Phase I SPH4336	
Drug	SPH4336 Tablets
Drug Type	Small molecule
Drug Class	CDK4/6 inhibitor
Dose	50-600 mg (tablets) per day
Route	Oral (p.o.)
Schedule of Administration	Once daily, continuous 21-day cycles
Patient Characteristics For Phase I SPH4336	
Number of Patients, Male	8
Number of Patients, Female	21
Stage	Stage III: 4
	Stage IV: 25
Age	Median (range), years: 55.0 (23-66)
Previous treatment with antitumor drugs, *n* (%)	27 (93.1)
Chemotherapy	25 (86.2)
Targeted therapy	14 (48.3)
Immunotherapy	6 (20.7)
Biotherapy	1 (3.4)
Others	18 (62.1)
Performance Status: ECOG, *n* (%)	
0	5 (17.2)
1	24 (82.8)
Median time from initial diagnosis	Months (range): 50.9 (4.7-140.2)
Cancer Types or Histologic Subtypes	
Breast cancer, *n* (%)	14 (48.3)
Sarcoma, *n* (%)	8 (27.6)
Liposarcoma, *n* (%)	4 (13.8)
Others	4 (13.8)
Non-small cell lung cancer, *n* (%)	2 (6.9)
Others, *n* (%)	5 (17.2)
Primary Assessment Method For Phase I SPH4336	
Number of Patients Enrolled	29
Number of Patients Evaluable for Toxicity	29
Number of Patients Evaluated for Efficacy	27
Evaluation Method	RECIST 1.1
Response Assessment CR	0%
Response Assessment PR	3.7%
Response Assessment SD	55.6%
Response Assessment PD	37.0%
(Median) Duration Assessments PFS	3.1 months; 95%CI: 1.7-5.7
(Median) Duration Assessments Duration of Treatment	4.1, 2.3, 1.5, 2.2, 1.5 and 2.0 months in 50-, 100-, 200-, 300-, 400- and 600-mg cohorts, respectively
**Assessment, analysis, and Discussion**	
Completion	Completed
Investigator’s Assessment	Drug tolerable, hints of efficacy

## Additional details of endpoints and study design

This phase I, open-label, single-arm, dose-escalation, and dose-expansion clinical trial was conducted in 4 hospitals in China to determine DLT, MTD, and RP2D of SPH4336 tablets in patients with advanced solid tumors. The study also aimed to characterize the PK parameters, anti-tumor activity, and safety profile of the study drug.

The key inclusion criteria included patients aged 18-70 years old with histologically or cytologically confirmed advanced solid tumors, refractory or intolerant to the standard treatment or no recommendation of standard treatment, an Eastern Cooperative Oncology Group (ECOG) performance status score of 0 or 1, life expectancy ≥3 months and measurable disease in the dose-expansion cohorts as per the Response Evaluation Criteria in Solid Tumours (RECIST) version 1.1. The key exclusion criteria included prior or current treatment with CDK4/6 inhibitors, any other anti-neoplastic therapy within 4 weeks, major surgery before or planned after the first dose, allergic constitution or history of severe allergy, active hepatitis B or C infection, history of immunodeficiency or mental disorders, the presence of factors that may cause prolongation of corrected QTc or arrhythmia, and severe cardiovascular and pulmonary diseases. This trial was conducted in accordance with the International Council for Harmonisation guidelines and applicable national laws and regulations along with the general ethical principles outlined in the Declaration of Helsinki.

During dose escalation, patients were first given a single dose of oral SPH4336 on the first day (cycle 0, day 1 [C0D1]). After a 7-day washout period, starting from cycle 1, day 1 (C1D1), the patients were administered continuous doses of SPH4336 once daily (QD) in 21-day cycles. DLTs were evaluated from C0D1 to C1D21 (28 days). DLT was defined as adverse events (AEs) associated with the treatment of SPH4336 that met any of the following criteria: (1) haematologic toxicity: grade 4 haematologic toxicity, grade ≥ 3 thrombocytopenia, febrile neutropenia and thromboembolism; (2) grade ≥ 3 non-haematologic toxicity, including grade ≥ 3 elevated total bilirubin (TBIL), grade ≥ 2 elevated TBIL with grade ≥ 2 elevated alanine aminotransferase (ALT) or aspartate aminotransferase (AST), grade ≥ 3 elevated ALT; (3) mean value of corrected QT interval (QTc) by Fredericia ≥ 501 milliseconds once in electrocardiogram (ECG) examination and confirmed after 15 minutes for the second time; (4) other clinically significant and intolerable exacerbations of laboratory measurements or clinical abnormalities compared with baseline.

Dose escalation of SPH4336 (50, 100, 200, 300, 400, or 600 mg) was performed using a traditional 3 + 3 design. Based on the safety profile and PK characteristics revealed from the dose-escalation period, 2 to 3 dose levels ≤ MTD (defined as the highest dose level at which <33% of patients had DLTs) or the preset highest dose level (600 mg QD) were selected for the dose-expansion period. Patients who benefited from the treatment as evaluated by investigators could continue treatment for a maximum of 12 months or until disease progression, intolerable toxicity, death, or discontinuation, as per the decision of the investigators and patients.

Safety was regularly monitored throughout the study period, including inquiries into patients’ conditions, physical examinations, vital signs, clinical laboratory tests, 12-lead ECG results, ECOG scores, and AEs. AEs were graded according to the Common Terminology Criteria for Adverse Events (version 5.0).

Tumour response was assessed every 6 weeks using either computerized tomography or magnetic resonance imaging scans, and the results were evaluated according to the RECIST version 1.1.^1^

Plasma concentrations of SPH3643 (the free alkali of SPH4336) were detected for PK analysis. Blood samples for a single-dose PK evaluation were collected pre-dose (within 30 minutes before administration) and at 1, 2, 4, 5, 6, 8, 10, 12, 24, 48, 72, 96, and 168 hours after administration. Blood samples for multi-dose PK evaluation were collected on cycle 1, day 13, (C1D13; pre-dose), cycle 1, day 14 (C1D14; pre-dose), cycle 1, day 15 (C1D15; pre-dose) at 1, 2, 4, 5, 6, 8, 10, 12, 24 hours after administration, cycle 2, day 1 (C2D1) to cycle 5, day 1 (C5D1), and day 1 of every 2 cycles (pre-dose).

All statistical analyses were performed using SAS 9.4 (SAS Institute Inc., Cary, North Carolina) except for analyses of PK parameters (Phoenix^®^ WinNonlin Professional 8.2, Pharsight Corp.). The Kaplan-Meier method was used to analyze the time-to-event variables.

## Outcome notes

### Patient characteristics and disposition

**Supplementary Table S1** summarizes the baseline characteristics of the 29 patients enrolled in the study between Nov 2020 and Feb 2023. The most common tumor types were breast cancer (*n* = 14, 48.3%) and sarcoma (*n* = 8, 27.6%). At the time of data cutoff (26 October 2023), all patients discontinued the study, mostly due to disease progression (*n* = 18, 62.1%). Death was reported in 1 patient due to disease progression (*n* = 1, 3.4%).

### Safety and tolerability

Of the 19 patients in whom SPH4336 was escalated from 50 to 600 mg, 1 patient (300 mg) was deemed invalid for DLT analysis due to inadequate drug administration caused by an AE unrelated to SPH4336. No DLT was observed, so MTD was not reached within 50-600 mg. The PK analysis of the 50-600-mg cohorts during the escalation phase showed no further increase in the internal exposure of SPH4336 from 400- to 600-mg cohort, indicating that drug absorption had reached saturation at 400 mg. Therefore, the 400 and 600 mg were set as the dose levels for the expansion cohorts. A total of 10 patients subsequently participated in the expansion phase with a dose level of 400 mg (*n* = 6) and 600 mg (*n* = 4) for further investigation.

All patients who were enrolled (*n* = 29) received at least 1 dose of the study drug and were included in the full analysis set (FAS) for safety evaluation. All patients experienced at least 1 treatment-related adverse event (TRAE). **Table 1** presents TRAEs that occurred in ≥10% of the total population. The most frequent hematologic TRAEs were decreased leucocyte count (*n* = 21, 72.4%), decreased neutrophil count (*n* = 20, 69.0%), and anemia (*n* = 19, 65.5%). The most frequent non-haematologic TRAEs were diarrhea (*n* = 20, 69.0%), vomiting (*n* = 12, 41.4%), abdominal pain and nausea (*n* = 9 each, 31.0%). The most common TRAEs of grade ≥ 3 were decreased leucocyte and lymphocyte count (*n* = 4 each, 13.8%) as well as decreased neutrophil count (*n* = 3, 10.3%). Of the 5 patients who reported serious adverse events (SAEs), only 1 in the 600-mg cohort had grade 3 increased γ-glutamyl transferase (GGT), which was considered to be related to the study treatment. Death was reported by 1 patient in the 200-mg cohort due to disease progression, which was considered unrelated to SPH4336.

### Pharmacokinetics

All 29 patients had at least 1 valid PK parameter and were included in the PK analysis. PK parameters after a single and a multiple dosing of SPH4336 are shown in **Supplementary Table S2** and the mean plasma concentration-time curve is shown in **Figure 1**. The exposure of SHP4336 (reflected by *C*_max_ and AUC_0-last_) increased proportionally with a dose ranging from 50 to 400 mg, but it did not increase from 400 to 600 mg. At doses of 50-600 mg, the median *T*_max_ was 10.0-47.8 hours, and the geometric mean terminal *t*_1/2_ was 52.8-72.3 hours after a single dose of SPH4336. After multiple dosing, SPH4336 was rapidly absorbed, with the median *T*_max_ ranging from 4.0 to 8.0 hours. The accumulation of SPH4336 was found after multiple doses of Rac_C_max_ and Rac_AUC, ranging from 2.22 to 4.32 and 2.29 to 5.43, respectively.

### Efficacy

A total of 27 patients (93.1%) who received ≥80% dose of the study drug in at least 1 cycle and had the tumor response assessment were included in the efficacy analysis set (ES). A patient with BC in the 400-mg cohort who lacked efficacy data and another patient with <80% of the designated dose during the multiple dosing phase were excluded from the ES.

The overall objective response rate (ORR; 95% CI) was 3.7% (0.1%-19.0%). By the cut-off date, there was 1 patient in the 600-mg cohort who achieved a confirmed partial response (PR) with a duration of response (DOR) of 5.6 months. A total of 15 (55.6%) patients across 50-600 mg cohorts had the best of response (BOR) with stable disease (SD). The overall DCR (95% CI) was 59.3% (38.8%-77.6%) in the ES population (**Supplementary Table S3**).

By the data cutoff, the endpoint events (PD or death) occurred in 20 patients (69.0%). The median progression free survival (PFS) (95% CI) was 3.1 months (1.7-5.7), and the PFS rates at 3, 6, 9, and 12 months were 53.9% (33.1%-70.8%), 29.4% (12.5%-48.6%), 29.4% (12.5%-48.6%) and 11.8% (2.1%-30.4%), respectively. The median PFS in the 50-mg cohort was not evaluable (NE) as neither PD nor death had occurred in more than half of the patients (**Supplementary Table S4**).

The depth of response (DPR), which is defined as the maximum percentage change in tumor size compared with baseline, was measured in 24 patients (82.8%) per RECIST v1.1 (**Supplementary Figure S1**). The best DPR was 3.5%, -3.9%, 11.3%, 5.0%, -11.5% and -34.6% in the 50-, 100-, 200-, 300-, 400- and 600-mg cohorts, respectively.

The efficacy of SPH4336 in the treatment of BC (*n* = 14) was separately analyzed. A total of 12 patients with BC were included in the ES population. The overall ORR was 8.3% (95% CI, 0.2%-38.5%). The patient who achieved confirmed PR was a patient with hormone receptor-positive (HR+) BC in the 600-mg cohort. A total of 6 patients (50.0%) had BOR with SD. The overall DCR was 58.3% (95% CI, 27.7%-84.8%), with a DCR (95% CI) of 100% (2.5%-100.0%), 57.1% (18.4%-90.1%) and 50.0% (6.8%-93.2%) in the 100-, 400- and 600-mg cohorts, respectively (**Supplementary Table S5**). The median PFS was 3.1 months (95% CI, 1.3-11.3), and the PFS rates at 3, 6, and 9 months were 58.7% (27.4%-80.4%), 35.2% (9.3%-63.3%) and 35.2% (9.3%-63.3%), respectively. The PFS rate at 12 months and the median PFS for the 100-mg cohort were not evaluable (NE) since the patient (*n* = 1) started a new anti-tumor medication before PD. The median PFS in the 400- and 600-mg cohorts were 2.4 (95% CI, 0.9-NE) and 3.1 (95% CI, 1.6-NE) months, respectively (**Supplementary Table S6**).

### Assessment, analysis, and discussion

In this study, the most frequent TRAEs (≥ 20%), mostly hematologic and gastrointestinal, were similar to the profiles of other CDK4/6 inhibitors.^2,3^ Most TRAEs were grade 1/2, which resolved or disappeared with or without treatment. Grade ≥ 3 TRAEs was comparable with the other phase I trials of CDK4/6 inhibitors, with the exception of increased GGT and hypokalaemia.^2-4^ The PALMIRA study showed the overall incidence of increased GGT was 4.4% in 135 patients treated with endocrine therapy combined with palbociclib; 3 patients (2.2%) and 1 patient (0.7%) had grade 3 and 4 increased GGT.^5^ In contrast, increased GGT was observed in only 1 of 60 patients (1.7%) treated solely with endocrine therapy and was categorized as grade < 3. The MONARCH 1 trial showed that 26.2% of patients (34 of 130) reported hypokalaemia of all grades treated with abemaciclib alone, while 2 patients reported hypokalaemia of grade 3.^6^ These findings suggest that CDK4/6 inhibitors may increase the risk of GGT abnormality and hypokalaemia, warranting ongoing monitoring in subsequent SPH4336 studies. Although SAEs were observed in 5 patients, TRAE was observed in only 1 patient with increased GGT. There was 1 death reported in the 200-mg cohort due to disease progression, which was not related to SPH4336.

PK assessments showed that SPH4336 exposure increased proportionally within the range of ~50-400 mg but did not increase further at 600 mg, suggesting absorption saturation at 400 mg QD. The ratio of *C*_trough_/IC_50_ for CDK4 and CDK6 of SPH4336 was comparable with abemaciclib^3,7^ and palbociclib^4^ at their FDA-approved dosing regimens (200 mg BID and 125 mg QD, respectively) (**Supplementary Table S7**), indicating that 400 mg QD of SPH4336 can effectively inhibit the activity of CDK4 and CDK6.

The overall ORR of 3.7% observed in this study was comparable to phase I trials of the other CDK4/6 inhibitors (**Supplementary Table S8**).^2,4,6,8^ Although dose-dependent, anti-tumor efficacy was not observed due to the small sample size, an analysis of PFS suggested that SPH4336 may be promising in the treatment of BC and sarcoma. The ORR and DCR of patients with HR + breast cancer (12.5% and 62.5%, respectively) were comparable to those of other CDK4/6 inhibitors.^6,9^

## Supplementary Material

oyaf077_suppl_Supplementary_Tables_1-8

oyaf077_suppl_Supplementary_Figures_1

## References

[1] Eisenhauer EA, Therasse P, Bogaerts J, et al New response evaluation criteria in solid tumours: revised RECIST guideline (version 1.1). Eur J Cancer. 2009;45:228-247. https://doi.org/10.1016/j.ejca.2008.10.026 19097774

[2] Infante JR, Cassier PA, Gerecitano JF, et al A phase i study of the cyclin-dependent kinase 4/6 inhibitor ribociclib (LEE011) in patients with advanced solid tumors and lymphomas. Clin Cancer Res. 2016;22:5696-5705. https://doi.org/10.1158/1078-0432.CCR-16-1248 27542767 PMC5621377

[3] Patnaik A, Rosen LS, Tolaney SM, et al Efficacy and safety of abemaciclib, an inhibitor of CDK4 and CDK6, for patients with breast cancer, non-small cell lung cancer, and other solid tumors. Cancer Discov. 2016;6:740-753. https://doi.org/10.1158/2159-8290.CD-16-0095 27217383

[4] Flaherty KT, Lorusso PM, Demichele A, et al Phase I, dose-escalation trial of the oral cyclin-dependent kinase 4/6 inhibitor PD 0332991, administered using a 21-day schedule in patients with advanced cancer. Clin Cancer Res. 2012;18:568-576. https://doi.org/10.1158/1078-0432.CCR-11-0509 22090362

[5] Llombart-Cussac A, Harper-Wynne C, Perello A, et al Second-line endocrine therapy (ET) with or without palbociclib (P) maintenance in patients (pts) with hormone receptor-positive (HR[+])/human epidermal growth factor receptor 2-negative (HER2[-]) advanced breast cancer (ABC): PALMIRA trial. J Clin Oncol. 2023;41:1001-1001. https://doi.org/10.1200/jco.2023.41.16_suppl.1001 PMC12169856 40294349

[6] Dickler MN, Tolaney SM, Rugo HS, et al MONARCH 1, a phase II study of abemaciclib, a CDK4 and CDK6 inhibitor, as a single agent, in patients with refractory HR^+^/HER2^−^ metastatic breast cancer. Clin Cancer Res. 2017;23:5218-5224. https://doi.org/10.1158/1078-0432.CCR-17-0754 28533223 PMC5581697

[7] Fujiwara Y, Tamura K, Kondo S, et al Phase 1 study of abemaciclib, an inhibitor of CDK 4 and 6, as a single agent for Japanese patients with advanced cancer. Cancer Chemother Pharmacol. 2016;78:281-288. https://doi.org/10.1007/s00280-016-3085-8 27312735

[8] Zhang J, Yang N, Ji D, et al A randomized phase i study of abemaciclib in chinese patients with advanced and/or metastatic cancers. Target Oncol. 2021;16:177-187. https://doi.org/10.1007/s11523-020-00789-9 33492568 PMC7935732

[9] Zhang P, Xu B, Gui L, et al A phase 1 study of dalpiciclib, a cyclin-dependent kinase 4/6 inhibitor in Chinese patients with advanced breast cancer. Biomark Res. 2021;9:24. https://doi.org/10.1186/s40364-021-00271-2 33845905 PMC8042970

