# Correction to: Phase I trial of SPH4336, a novel cyclin-dependent kinase 4/6 inhibitor, in patients with advanced solid tumors

**DOI:** 10.1093/oncolo/oyaf366

**Published:** 2026-01-29

This is a correction to: Yu Jiang, Xu Liang, Mei-Li Sun, Ge Gao, Yi Gong, Hui-Ping Li, Jie Liu, Yong-Sheng Wang, Phase I trial of SPH4336, a novel cyclin-dependent kinase 4/6 inhibitor, in patients with advanced solid tumors, *The Oncologist*, Volume 30, Issue 6, June 2025, https://doi.org/10.1093/oncolo/oyaf077

The following changes have been made to the originally ­published manuscript. The affiliations for the 4th and 5th authors have been corrected. These now read: “Ge Gao^1^” instead of: “Ge Gao^4^”; and “Yi Gong^4^” instead of: “Yi Gong^1^”.

The emendations have been made to the article.

