# Supplementary material for: Phase I trial of SPH4336, a novel cyclin-dependent kinase 4/6 inhibitor, in patients with advanced solid tumors
Source: Oncologist. 2025 Jun 30;30(6):oyaf077. doi: 10.1093/oncolo/oyaf077 (PMC12207880; doi:10.1093/oncolo/oyaf077)
Supplement: oyaf077_suppl_Supplementary_Tables_1-8 [file oyaf077_suppl_supplementary_tables_1-8.docx]

**Supplementary Tables**

**Supplementary Table 1**. Patient characteristics at baseline.

| Characteristics | 50 mg (N=3) | 100 mg (N=3) | 200 mg (N=3) | 300 mg (N=4) | 400 mg (N=9) | 600 mg (N=7) | All (N=29) |
| --- | --- | --- | --- | --- | --- | --- | --- |
| Median age, years (range) | 51.0 (49-52) | 55.0 (54-59) | 48.0 (42-61) | 59.0 (46-60) | 61.0 (39-66) | 45.0 (23-56) | 55.0 (23-66) |
| Sex |  |  |  |  |  |  |  |
| Male, n (%) | 2 (66.7) | 2 (66.7) | 1 (33.3) | 1 (25.0) | 1 (11.1) | 1 (14.3) | 8 (27.6) |
| Female, n (%) | 1 (33.3) | 1 (33.3) | 2 (66.7) | 3 (75.0) | 8 (88.9) | 6 (85.7) | 21 (72.4) |
| ECOG performance status, n (%) |  |  |  |  |  |  |  |
| 0 | 0 | 0 | 1 (33.3) | 1 (25.0) | 3 (33.3) | 0 | 5 (17.2) |
| 1 | 3 (100.0) | 3 (100.0) | 2 (66.7) | 3 (75.0) | 6 (66.7) | 7 (100.0) | 24 (82.8) |
| Median time since first diagnosis, months (range) | 11.6 (11.6-11.6) | 138.4 (138.4-138.4) | 22.2 (22.2-22.2) | 26.6 (10.9-129.6) | 56.3 (41.4-140.2) | 52.8 (4.7-124.8) | 50.9 (4.7-140.2) |
| Primary tumour type, n (%) |  |  |  |  |  |  |  |
| Breast cancer | 0 | 1 (33.3) | 0 | 0 | 9 (100.0) | 4 (57.0) | 14 (48.3) |
| Sarcoma | 1 (33.3) | 2 (66.7) | 1 (33.3) | 2 (50.0) | 0 | 2 (28.6) | 8 (27.6) |
| Liposarcoma | 1 (33.3) | 0 | 1 (33.3) | 1 (25.0) | 0 | 1 (14.3) | 4 (13.8) |
| Others | 0 | 2 (66.7) | 0 | 1 (25.0) | 0 | 1 (14.3) | 4 (13.8) |
| Non-small cell lung cancer | 0 | 0 | 1 (33.3) | 1 (25.0) | 0 | 0 | 2 (6.9) |
| Others | 2 (66.7) | 0 | 1 (33.3) | 1 (25.0) | 0 | 1 (14.3) | 5 (17.2) |
| Tumour stage at study entry |  |  |  |  |  |  |  |
| III | 1 (33.3) | 0 | 1 (33.3) | 1 (25.0) | 0 | 1 (14.3) | 4 (13.8) |
| IV | 2 (66.7) | 3 (100.0) | 2 (66.7) | 3 (75.0) | 9 (100.0) | 6 (85.7) | 25 (86.2) |
| Previous treatment with anti-tumour drugs, n (%) | 2 (66.7) | 2 (66.7) | 3 (100.0) | 4 (100.0) | 9 (100.0) | 7 (100.0) | 27 (93.1) |
| Chemotherapy | 1 (33.3) | 2 (66.7) | 3 (100.0) | 4 (100.0) | 9 (100.0) | 6 (85.7) | 25 (86.2) |
| Targeted therapy | 2 (66.7) | 1 (33.3) | 3 (100.0) | 2 (50.0) | 4 (44.4) | 2 (28.6) | 14 (48.3) |
| Immunotherapy | 2 (66.7) | 0 | 1 (33.3) | 1 (25.0) | 2 (22.2) | 0 | 6 (20.7) |
| Biotherapy | 0 | 0 | 0 | 0 | 1 (11.1) | 0 | 1 (3.4) |
| Others | 0 | 1 (33.3) | 2 (66.7) | 1 (25.0) | 9 (100.0) | 5 (71.4) | 18 (62.1) |

**Supplementary Table 2**. Pharmacokinetic parameters of SPH3643 after a single and multiple dosing of SPH4336.

|  | **50 mg** | **100 mg** | **200 mg** | **300 mg** | **400 mg** | **600 mg** |
| --- | --- | --- | --- | --- | --- | --- |
| **Single dosing** | **n=3** | **n=3** | **n=3** | **n=4** | **n=8** | **n=6** |
| C_max_ (ng/mL) | 4.35 (35.9%) | 7.71 (19.9%) | 13.5 (58.3%) | 14.7 (21.7%) | 32.2 (53.9%) | 27.4 (17.1%) |
| T_max_ (h) | 10.00 (5.00, 10.00) | 11.97 (5.00, 23.93) | 47.78 (12.00, 48.03) | 14.97 (5.00, 48.13) | 12.09 (10.00, 48.05) | 23.86 (12.00, 24.03) |
| AUC_0-last_ (ngхh/mL) | 254 (37.0%) | 618 (14.3%) | 1368 (48.0%) | 1429 (28.6%) | 3175 (48.5%) | 2865 (14.9%) |
| AUC_0-24_ (ng х h/mL) | 72.6 (19.8%) | 143 (4.0%) | 231 (59.8%) | 244 (31.1%) | 577 (52.9%) | 495 (16.5%) |
| AUC_0-inf_ (ng х h/mL) | NC | 712 (14.2%) | 1597 (47.0%) | 2096 | 5123 (2160, 8086) | 3085 (2912, 3259) |
| MRT_0-inf_ (h) | NC | 85.7 (8.1%) | 92.1 (13.3%) | 102 | 88.0 (75.9, 100) | 102 (100, 104) |
| CLz/F (L/h) | NC | 140 (14.2%) | 125 (47.0%) | 143 | 117 (49.5, 185) | 195 (184, 206) |
| Vz/F (L) | NC | 11193 (21.7%) | 9542 (51.0%) | 14934 | 10074 (3528, 16620) | 17403 (16078, 18729) |
| _t1/2_ (h) | NC | 55.3 (15.1%) | 52.8 (11.5%) | 72.3 | 55.8 (49.4, 62.2) | 61.8 (60.5, 63.0) |
| **Multiple dosing** | **n=3** | **n=3** | **n=3** | **n=3** | **n=7** | **n=7** |
| C_max_ (ng/mL) | 18.5 (8.3%) | 26.4 (12.4%) | 42.8 (85.2%) | 65.4 (22.0%) | 74.0 (23.1%) | 62.7 (15.4%) |
| T_max_ (h) | 8.00 (8.00, 11.95) | 5.00 (5.00, 8.05) | 8.00 (4.00, 8.00) | 4.00 (4.00, 5.00) | 5.00 (4.00, 10.00) | 4.00 (1.92, 7.98) |
| AUC_0-tau_ (ng·h/mL) | 394 (5.6%) | 520 (15.1%) | 1077 (420, 1734) | 1231 (23.2%) | 1420 (27.5%) | 1323 (15.6%) |
| C_trough_ (ng/mL) | 13.3 (3.0%) | 17.8 (19.8%) | 35.7 (96.0%) | 45.8 (18.3%) | 58.2 (37.5%) | 50.8 (16.2%) |
| CLss/F (L/h) | 127 (5.6%) | 192 (15.1%) | 296 (115, 476) | 244 (23.2%) | 282 (27.5%) | 453 (15.6%) |
| Rac_C_max_ | 4.26 (27.4%) | 3.42 (32.6%) | 3.18 (21.6%) | 4.32 (15.0%) | 2.25 (42.0%) | 2.22 (23.1%) |
| Rac_AUC | 5.43 (14.1%) | 3.62 (17.3%) | 3.79 (3.20, 4.38) | 4.76 (20.5%) | 2.29 (33.7%) | 2.61 (25.9%) |
| DF (%) | 33.4 (26.3%) | 39.0 (27.4%) | 28.2 (26.3, 30.2) | 41.6 (16.7%) | 28.3 (56.4%) | 23.3 (18.0%) |

NC: incalculable. T_max_: time to maximum plasma concentration; C_max_: maximum plasma concentration; AUC_0-last_: area under the concentration-time curve (AUC) from dosing to the time of the last measured concentration; AUC_0-tau_: AUC over the dosing interval; AUC_0-inf_: AUC from dosing to infinity; C_trough_: trough plasma concentration at steady-state; Rac_AUC_0-tau_ and Rac_C_max_: accumulation ratio; t_1/2_: elimination half-life; CL/F: apparent plasma clearance of drug after extravascular administration; Vz/F: apparent volume of distribution after extravascular administration.

When AUC_%Extrap_ (%) was >20%, AUC_0-inf_, CLz/F, Vz/F, λz and T_1/2_ were excluded from statistical analyses of PK parameters. T_max_ was displayed as median (minimum, maximum), and the others were represented as the geometric mean (relative geometric variation %) when at least 3 values were evaluable. If only 2 or 1 value(s) were evaluable, the mean (minimum, maximum) or the single value was displayed, respectively.

The PK parameters of SPH3643 in the single-dosing phase were not calculated for 2 patients (1 in the 400-mg cohort due to a concentration of higher than 5% C_max_ before C0D1 single dosing; the other in the 600-mg cohort who were wrongly given 100 mg on C0D1 single dosing). Due to early withdrawal from the study, pharmacokinetic parameters after multiple dosing were not obtained for 3 patients (1 in the 300-mg cohort and 2 in the 400-mg cohort groups).

**Supplementary Table 3**. Tumour response per RECIST v1.1 (ES)

|  | **50 mg** | **100 mg** | **200 mg** | **300 mg** | **400 mg** | **600 mg** | **All** |
| --- | --- | --- | --- | --- | --- | --- | --- |
|  | **(N=3)** | **(N=3)** | **(N=3)** | **(N=4)** | **(N=7)** | **(N=7)** | **(N=27)** |
| Best overall response, n (%) |  |  |  |  |  |  |  |
| CR | 0 | 0 | 0 | 0 | 0 | 0 | 0 |
| PR | 0 | 0 | 0 | 0 | 0 | 1 (14.3) | 1 (3.7) |
| SD | 3 (100) | 2 (66.7) | 1 (33.3) | 2 (50.0) | 4 (57.1) | 3 (42.9) | 15 (55.6) |
| PD | 0 | 1 (33.3) | 2 (66.7) | 2 (50.0) | 3 (42.9) | 2 (28.6) | 10 (37.0) |
| NE | 0 | 0 | 0 | 0 | 0 | 1 (14.3) | 1 (3.7) |
|  |  |  |  |  |  |  |  |
| ORR, % (95% CI) | 0 (0.0, 70.8) | 0 (0.0, 70.8) | 0 (0.0, 70.8) | 0 (0.0, 60.2) | 0 (0.0, 41.0) | 14.3 (0.4, 57.9) | 3.7 (0.1, 19.0) |
| *DCR, % (95% CI) | 100.0 (29.2,100) | 66.7 (9.4,99.2) | 33.3 (0.8,90.6) | 50.0 (6.8,93.2) | 57.1 (18.4,90.1) | 57.1 (18.4,90.1) | 59.3 (38.8,77.6) |

NE: non-evaluable.

*Patients who achieved confirmed CR, PR or SD sustained for 35 days throughout the study process from enrolment to disease progression.

**Supplementary Table 4.** Kaplan–Meier analysis for PFS (FAS)

|  | **50 mg** | **100 mg** | **200 mg** | **300 mg** | **400 mg** | **600 mg** | **All** |
| --- | --- | --- | --- | --- | --- | --- | --- |
|  | **(N=3)** | **(N=3)** | **(N=3)** | **(N=4)** | **(N=9)** | **(N=7)** | **(N=29)** |
| Endpoint events, n (%) |  |  |  |  |  |  |  |
| PD, n (%) | 1 (33.3%) | 2 (66.7%) | 3 (100%) | 4 (100%) | 6 (66.7%) | 4 (57.1%) | 20 (69.0%) |
| Death, n (%) | 0 | 0 | 0 | 0 | 0 | 0 | 0 |
| Censored subjects | 2 (66.7%) | 1 (33.3%) | 0 | 0 | 3 (33.3%) | 3 (42.9%) | 9 (31.0%) |
| PFS (month) |  |  |  |  |  |  |  |
| Minimum–Maximum* | 2.83^+^ - 31.97^+^ | 1.71 - 9.72 | 0.76 - 5.59 | 1.74 - 5.75 | 0.03^+^ - 11.37^+^ | 0.89^+^ - 11.33 | 0.03^+^ - 31.97^+^ |
| Median, % (95% CI) | NE (4.2, NE) | 9.7 (1.7, NE) | 1.6 (0.8, NE) | 2.4 (1.7, NE) | 2.4 (0.9, NE) | 3.1 (1.5, NE) | 3.1 (1.7, 5.7) |
| PFS rate |  |  |  |  |  |  |  |
| 3 months, % (95% CI) | 100 (100, 100) | 66.7 (5.4, 94.5) | 33.3 (0.9, 77.4) | 25.0 (0.9, 66.5) | 50.0 (15.2, 77.5) | 66.7 (19.5, 90.4) | 53.9 (33.1, 70.8) |
| 6 months, % (95% CI) | 50.0 (0.6, 91.0) | 66.7 (5.4, 94.5) | 0.0 (NE, NE) | 0.0 (NE, NE) | 33.3 (5.6, 65.8) | 44.4 (6.6, 78.5) | 29.4 (12.5, 48.6) |
| 9 months, % (95% CI) | 50.0 (0.6, 91.0) | 66.7 (5.4, 94.5) | 0.0 (NE, NE) | 0.0 (NE, NE) | 33.3 (5.6, 65.8) | 44.4 (6.6, 78.5) | 29.4 (12.5, 48.6) |
| 12 months, % (95% CI) | 50.0 (0.6, 91.0) | 0.0 (NE, NE) | 0.0 (NE, NE) | 0.0 (NE, NE) | NE (NE, NE) | 0.0 (NE, NE) | 11.8 (2.1, 30.4) |

NE: unevaluable.

* Kaplan–Meier method was used for estimation. ^+^ was used to represent any censored value in calculation of minimum and maximum values.

**Supplementary Table 5.** Tumour response of breast cancer per RECIST v1.1

|  | **50 mg** | **100 mg** | **200 mg** | **300 mg** | **400 mg** | **600 mg** | **All** |
| --- | --- | --- | --- | --- | --- | --- | --- |
|  | **(N=0)** | **(N=1)** | **(N=0)** | **(N=0)** | **(N=7)** | **(N=4)** | **(N=12)** |
| Best overall response, n (%) |  |  |  |  |  |  |  |
| CR | 0 | 0 | 0 | 0 | 0 | 0 | 0 |
| PR | 0 | 0 | 0 | 0 | 0 | 1 (25.0) | 1 (8.3) |
| SD | 0 | 1 (100) | 0 | 0 | 4 (57.1) | 1 (25.0) | 6 (50.0) |
| PD | 0 | 0 | 0 | 0 | 3 (42.9) | 1 (25.0) | 4 (33.3) |
| NE | 0 | 0 | 0 | 0 | 0 | 1 (25.0) | 1 (8.3) |
|  |  |  |  |  |  |  |  |
| ORR, % (95%CI) | 0 (NE, NE) | 0 (0.0, 97.5) | 0 (NE, NE) | 0 (NE, NE) | 0 (0.0, 41.0) | 25.0 (0.6, 80.6) | 8.3 (0.2, 38.5) |
| *DCR, % (95%CI) | 0 (NE, NE) | 100(2.5, 100) | 0(NE, NE) | 0(NE, NE) | 57.1 (18.4, 90.1) | 50.0 (6.8, 93.2) | 58.3 (27.7, 84.8) |

NE: inevaluable.

*Patients who achieved confirmed CR, PR or SD sustained for 35 days throughout the study process from enrolment to disease progression.

**Supplementary Table 6.** Kaplan–Meier analysis of breast cancer for PFS

|  | **50 mg** | **100 mg** | **200 mg** | **300 mg** | **400 mg** | **600 mg** | **All** |
| --- | --- | --- | --- | --- | --- | --- | --- |
|  | **(N=0)** | **(N=1)** | **(N=0)** | **(N=0)** | **(N=9)** | **(N=4)** | **(N=14)** |
| Endpoint events, n (%) |  |  |  |  |  |  |  |
| PD, n (%) | 0 | 0 | 0 | 0 | 6 (66.7) | 3 (75.0) | 9 (64.3) |
| Death, n (%) | 0 | 0 | 0 | 0 | 0 | 0 | 0 |
| Censored subjects | 0 | 1 (100%) | 0 | 0 | 3 (33.3) | 1 (25.0) | 5 (35.7) |
| PFS (month) |  |  |  |  |  |  |  |
| Minimum - Maximum* |  | 1.81^+^ - 1.81^+^ |  |  | 0.03^+^ - 11.37^+^ | 0.89^+^ - 11.33 | 0.03^+^ - 11.37^+^ |
| Median, % (95%CI) |  | NE (NE, NE) |  |  | 2.4 (0.9, NE) | 3.1 (1.6, NE) | 3.1 (1.3, 11.3) |
| PFS rate |  |  |  |  |  |  |  |
| 3 months, % (95%CI) |  | NE (NE, NE) |  |  | 50.0 (15.2, 77.5) | 66.7 (5.4, 94.5) | 58.7 (27.4, 80.4) |
| 6 months, % (95%CI) |  | NE (NE, NE) |  |  | 33.3 (5.6, 65.8) | 33.3 (0.9, 77.4) | 35.2 (9.3, 63.3) |
| 9 months, % (95%CI) |  | NE (NE, NE) |  |  | 33.3 (5.6, 65.8) | 33.3 (0.9, 77.4) | 35.2 (9.3, 63.3) |
| 12 months, % (95%CI) |  | NE (NE, NE) |  |  | NE (NE, NE) | 0.0 (NE, NE) | NE (NE, NE) |

NE: unevaluable.

* Kaplan–Meier method was used for estimation. ^+^ was used to represent any censored value in the calculation of minimum and maximum values.

**Supplementary Table 7.** Comparison of C_trough_/IC_50_ between SPH4336 and other CDK4/6 inhibitors

| PK parameter | SPH4336 | | Abemaciclib | | | Palbociclib | |
| --- | --- | --- | --- | --- | --- | --- | --- |
|  | CDK4 | CDK6 | CDK4 | CDK6 | | CDK4 | CDK6 |
| IC_50_ (nM) | 2.2 | 8.3 | 2.6 | 23 | | 3.7 | 12 |
| IC_50_ (ng/mL) | 1.1 | 4.2 | 1.3 | 11.7 | | 1.7 | 5.4 |
| IC_50_(ng/mL, ppb adjusted) | 9.5 | 35.8 | 35.6 | 314.9 | | 11.3 | 36.5 |
|  | 400 mg QD | | 200 mg BID | | | 125 mg QD | |
| C_trough_ (ng/mL) | 58.2 (37.5%, n=7) | | 197 (82%, n=52) | | | 47 (48%, n=4) | |
|  |  |  | 210 (89%, n=5) | | |  |  |
| C_trough_/IC_50_  ppb adjusted | 6.1 | 1.6 | 5.5 | | 0.6 | 4.2 | 1.3 |
|  |  |  | 5.9 | | 0.7 |  |  |
| ppb: plasma protein binding. | | | | | | | |

**Supplementary Table 8.** Efficacy of other CDK4/6 inhibitors

|  | **Palbociclib** | **Ribociclib** | **Abemaciclib** | **Abemaciclib** |
| --- | --- | --- | --- | --- |
| Sample size | 37 | 132 | 33* | 25 |
| region | U.S.A | U.S.A, France, Netherland | U.S.A, | China |
| Overall ORR (%) | 0% | 2.3% | 0% | 8.0% |
| Overall DCR (%) | 35% | 32.6% | 48.5% | 68.0% |
| *Data of the escalation phase were shown. | | | | |
