# Supplementary figures and images for: Phase I trial of SPH4336, a novel cyclin-dependent kinase 4/6 inhibitor, in patients with advanced solid tumors
Source: Oncologist. 2025 Jun 30;30(6):oyaf077. doi: 10.1093/oncolo/oyaf077 (PMC12207880; doi:10.1093/oncolo/oyaf077)

**
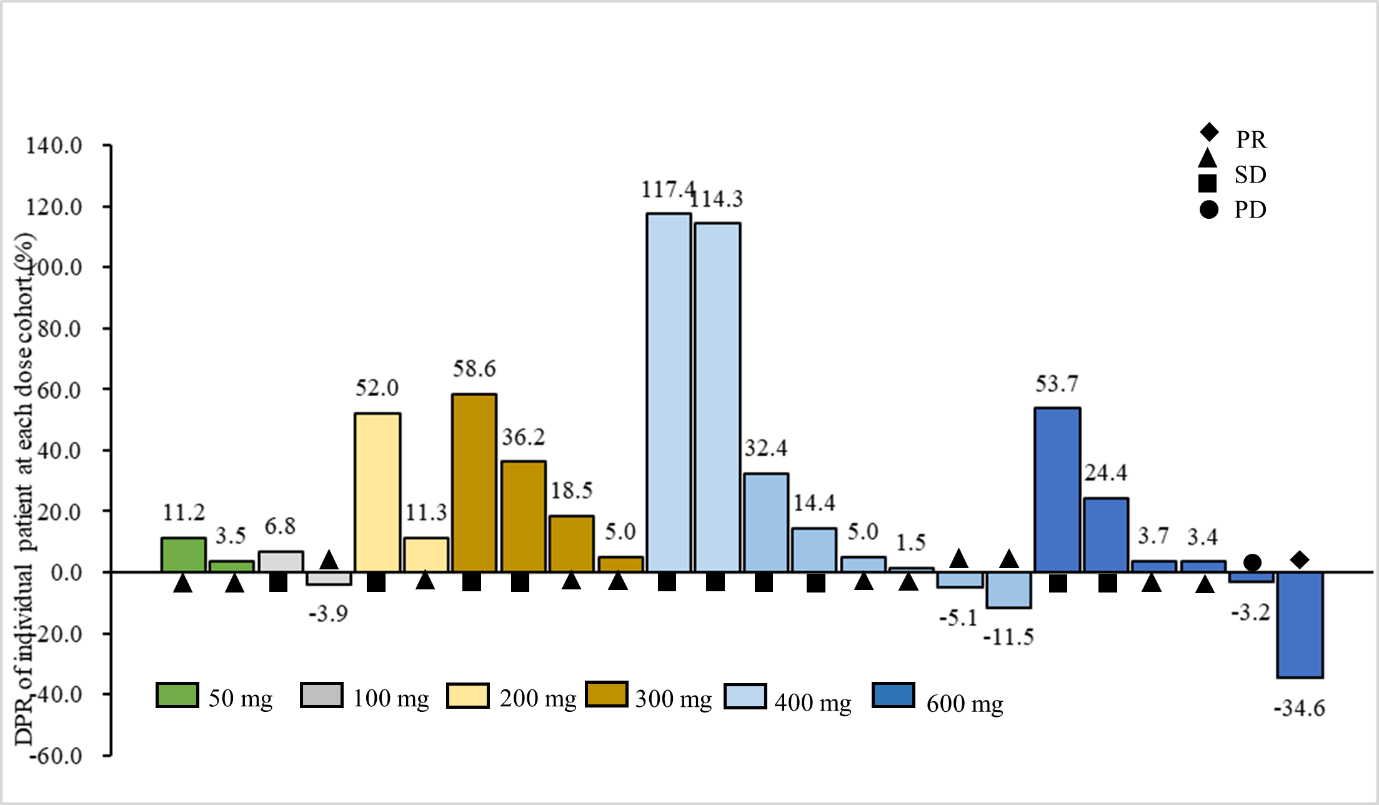
**

**Supplementary Figure 1.** Water-fall plot of best deepness of response (DPR) at each dose cohort.

Supplement: oyaf077_suppl_Supplementary_Figures_1 [file oyaf077_suppl_supplementary_figures_1.docx]
